# Recurrence and prognosis in intrahepatic cholangiocarcinoma patients with different etiology after radical resection: a multi-institutional study

**DOI:** 10.1186/s12885-022-09448-w

**Published:** 2022-03-26

**Authors:** Qi Li, Chen Chen, Jingbo Su, Yinghe Qiu, Hong Wu, Tianqiang Song, Xianhai Mao, Yu He, Zhangjun Cheng, Jingdong Li, Wenlong Zhai, Dong Zhang, Zhimin Geng, Zhaohui Tang

**Affiliations:** 1 Department of Hepatobiliary Surgery, The First Affiliated Hospital of Xi’an Jiaotong University, Xi’an, 710061 Shaanxi China; 2 Department of Biliary Surgery, Oriental Hepatobiliary Hospital Affiliated to Naval Medical University, Shanghai, 200433 China; 3 Department of Hepatobiliary and Pancreatic Surgery, West China Hospital of Sichuan University, Chengdu, 610041 China; 4 Department of Hepatobiliary Oncology, Tianjin Medical University Cancer Hospital, Tianjin, 300060 China; 5 Department of Hepatobiliary Surgery, Hunan Provincial People’s Hospital, Changsha, 410005 China; 6 Department of Hepatobiliary Surgery, The First Hospital Affiliated to Army Medical University, Chongqing, 400038 China; 7 Department of Hepatobiliary Surgery, Zhongda Hospital of Southeast University, Nanjing, 210009 China; 8 Department of Hepatobiliary Surgery, Affiliated Hospital of North Sichuan Medical College, Nanchong, 637000 China; 9 Hepatobiliary Pancreas and Liver Transplantation Surgery, The First Affiliated Hospital of Zhengzhou University, Zhengzhou, 450052 China; 10 Department of General Surgery, Xinhua Hospital Affiliated to Shanghai Jiaotong University School of Medicine, Shanghai, 200092 China

**Keywords:** Intrahepatic cholangiocarcinoma, Hepatitis B virus, Hepatolithiasis, Recurrence, Prognosis

## Abstract

**Objective:**

We aimed to evaluate the prognosis and adjuvant chemotherapy (ACT) in intrahepatic cholangiocarcinoma (ICC) patients with different etiology after radical resection.

**Methods:**

A total of 448 patients with ICC who underwent radical resection between 2010 and 2018 at ten Chinese tertiary hospitals were analyzed in the study. These patients were divided into conventional ICC (Con-ICC, *n* = 261, 58.2%), hepatitis B virus ICC (HBV-ICC, *n* = 102, 22.8%) and hepatolithiasis (Stone-ICC, *n* = 85,19.0%) subtypes according to different etiology. Propensity score matching (PSM) was conducted to mitigate the baseline differences between Con-ICC and HBV-ICC, Con-ICC and Stone-ICC, HBV-ICC and Stone-ICC subtypes.

**Results:**

Univariate and multivariate analysis showed that different etiology was a prognostic factor for overall survival and relapse-free survival, and different etiology was an independent risk factor for overall survival in ICC patients, respectively (*P* < 0.05). In addition, there was a statistical difference for overall survival in early recurrence patients among the three etiological subtypes (*P* < 0.05). After PSM, the overall survival of patients with Stone-ICC was worse than those of Con-ICC and HBV-ICC subtypes (*P* < 0.05), while the relapse-free survival of patients with Stone-ICC was equivalent to patients with Con-ICC and HBV-ICC (*P* > 0.05). In Stone-ICC patients, the median overall survival was 16.0 months and 29.7 months, and the median relapse-free survival was 9.0 months and 20.0 months for non-ACT and ACT patients, respectively (*P* < 0.05).

**Conclusion:**

The prognosis of Stone-ICC patients was significantly worse than those of Con-ICC and HBV-ICC patients. Interestingly, postoperative adjuvant chemotherapy can improve the prognosis of Stone-ICC patients effectively.

**Supplementary Information:**

The online version contains supplementary material available at 10.1186/s12885-022-09448-w.

## Introduction

Intrahepatic cholangiocarcinoma (ICC), originating above the secondary branches of the bile duct, is the second most common biliary malignancy and accounts for about 10% to 15% of primary liver carcinoma [1, 2]. In recent years, the incidence of ICC has shown a significant upward trend worldwide [3, 4]. Surgical resection is the only potentially curative treatment option for ICC patients, but only 20% of the first-diagnosed patients are eligible for radical resection [5]. In addition, the prognosis of ICC patients is very poor, and the median overall survival (OS) and relapse-free survival (RFS) are about 30 months and 20 months, and the 5-year survival rate is approximately 30%, respectively [6, 7]. Besides, the benefits of postoperative adjuvant chemotherapy (ACT) are still unclear for ICC patients and need further research [8, 9].

Currently, the pathogenic factors of ICC are geographically different, mainly including primary sclerosing cholangitis (PSC), Caroli disease, cirrhosis, non-alcoholic fatty liver disease (NLFLD), hepatitis B virus (HBV) infection, intrahepatic bile duct stones (hepatolithiasis), and liver flukes [10–13]. According to the epidemiological data from China, hepatolithiasis and HBV infection are the most important risk factors for the occurrence of ICC, account for 15 ~ 30% of ICC patients with hepatolithiasis and 30 ~ 50% with HBV infection, respectively [14–17]. Different etiological subtypes have variable pathological characteristics, leading to the differences on prognosis of ICC patients. However, the differences on OS and RFS, as well as the benefits of ACT on prognosis of the three etiological subtypes for ICC patients are still unclear. The study aimed to evaluate the prognosis and the benefits of ACT in ICC patients with different etiology after radical resection.

## Methods

### Patients and design

All patients undergoing curative resection for histologically confirmed ICC between 2010 and 2018 at ten tertiary hospitals in China (Oriental Hepatobiliary Hospital Affiliated to Naval Medical University; West China Hospital of Sichuan University; Tianjin Medical University Cancer Institute and Hospital; Hunan Provincial People’s Hospital; The First Hospital Affiliated to Army Medical University; Zhongda Hospital of Southeast University; The First Affiliated Hospital of Xi’an Jiaotong University; Affiliated Hospital of North Sichuan Medical College; Xinhua Hospital Affiliated to Shanghai Jiaotong University School of Medicine; The First Affiliated Hospital of Zhengzhou University) were considered for inclusion.

The inclusion criteria were as follows: (1) patients underwent radical resection and margin status recorded microscopically negative (R0); (2) patients with HBV infection (HBsAg ( +) and /or HBcAb ( +)); (3) patients with bile duct stones in histology; (4) the data of clinicopathological characteristics and follow-up data were all available. The exclusion criteria were as follows: (1) the history of HBV infection and hepatolithiasis was not recorded in detail; (2) patients combined with HCV infection or HCV + HBV infection or HBV + hepatolithiasis or HCV + hepatolithiasis before surgery; (3) patients combined with PSC or Caroli disease in histology; (4) patients died within 30 days after surgery. In this study, ICC only with HBV infection (HBsAg ( +) and /or HBcAb ( +)) was defined as HBV-ICC, ICC only with hepatolithiasis was defined as Stone-ICC, and ICC with no identifiable cause (such as HBV, HCV, hepatolithiasis, PSC, Caroli disease, NLFLD, etc.) was defined as Con-ICC. Finally, a total of 448 patients were included in the study after the strict inclusion and exclusion criteria. All included patients were evaluated according to the 8^th^ edition AJCC staging system and were followed up through December 2020.

### The regimens of postoperative ACT

Patients with postoperative ACT were strictly performed as follows. The regimens of included gemcitabine (1000 mg/m^2^ on days 1 and 8) + capecitabine (1250 mg/m^2^ twice daily on days 1–14) of a three-week cycle; gemcitabine (1000 mg/m^2^ on days 1 and 8) + cisplatin (30 mg/m^2^ on days 1 and 8) of a three-week cycle; gemcitabine (1000 mg/m^2^ on days 1 and 8) + oxaliplatin (100 mg/m^2^ on day 1) of a three-week cycle; gemcitabine (1000 mg/m^2^ on days 1 and 8) + tegafur (40 ~ 60 mg twice daily on days 1–14) of a three-week cycle.

In this study, the inclusion criteria for ACT included T_2~4_ stage, N1 stage, combined with major vascular invasion, or microvascular invasion, or perineural invasion, which was associated with high postoperative recurrence risk, and the selection of the chemotherapy regimen was mainly based on the ASCO clinical practice guideline [18], and whether the patient was tolerant to the drug. There were 166 patients with ICC who received ACT. Among the 90 Con-ICC patients, 25 (27.8%) patients received gemcitabine + capecitabine, 27 (30.0%) patients received gemcitabine + cisplatin, 30 (33.3%) patients received gemcitabine + oxaliplatin, and 8 (8.9%) patients received gemcitabine + tegafur, respectively. Among the 45 HBV-ICC patients, 12 (26.7%) patients received gemcitabine + capecitabine, 16 (35.5%) patients received gemcitabine + cisplatin, 14 (31.1%) patients received gemcitabine + oxaliplatin, and 3 (6.7%) patients received gemcitabine + tegafur, respectively. Among the 31 Stone-ICC patients, 6 (19.4%) patients received gemcitabine + capecitabine, 14 (45.2%) patients received gemcitabine + cisplatin, and 11 (35.4%) patients received gemcitabine + oxaliplatin, respectively.

### Follow-up

All patients included in the study were followed up after surgery. Routine follow-up was performed in outpatient and telephone. Liver function, tumor biomarkers (CEA, CA19-9, CA125), and ultrasound, contrast-enhanced CT or MRI examination were reviewed every 2 to 3 months within 1 year after surgery, and follow-up was conducted once every 3–6 months for more than 1 year after surgery. Recurrence referred to the discovery of new lesions by two or more imaging examinations. OS and RFS were calculated from the date of radical resection until the date of the most recent follow-up or death of the patients, and clinical evidence of tumor recurrence, respectively.

### Propensity score matching

Propensity score matching (PSM), as a very practical, novel, and creative statistical method for evaluating intervention effects using non-randomized controlled data, was conducted to mitigate the baseline differences affecting long-term outcomes between Con-ICC and HBV-ICC, Con-ICC and Stone-ICC, HBV-ICC and Stone-ICC subtypes [19], which was conducted by SPSS version 25 (IBM Corp., Armonk, NY, USA). Propensity score analysis with 1:1 matching was performed within a range of 0.02 of standard deviation.

### Statistical analysis

All statistical analyses were performed using SPSS version 25. Categorical variables were examined using χ^2^ test. The Kaplan–Meier method and Log-rank test were conducted for univariate analysis, and Cox proportional hazard regression model was conducted for multivariate analysis. The Kaplan–Meier curves and histograms were conducted by GraphPad Prism (version 8.0, San Diego, California, USA). Variables with *P* < 0.05 were considered as statistically significant.

## Results

### Comparison of clinicopathological characteristic for different etiological subtypes in ICC Patients

A total of 448 patients undergoing radical resection for histologically confirmed ICC between 2010 and 2018 were considered for inclusion. The comparison of clinicopathological characteristic of Con-ICC (no identifiable cause, *n* = 261, 58.2%), HBV-ICC (*n* = 102, 22.8%) and Stone-ICC (*n* = 85, 19.0%) was summarized in Table 1. The three etiological subtypes in ICC patients had a certain correlation with clinicopathological characteristics of sex, age (year), obstructive jaundice, CA19-9, Child–Pugh grade, tumor location, morphologic grape, perineural invasion, liver capsule involvement, and AJCC 8th edition N stage (*P* < 0.05). In addition, Stone-ICC had a higher proportion of CA19-9 > 39.0 U/ml, presence of perineural invasion, and morphologic grape with periductal infiltrating and intraductal growth compared with HBV-ICC (*P* < 0.05).

**Table 1:** Comparison of clinicopathological characteristic for ICC with different etiological subtypes after radical resection

	ICC with different etiological subtypes			*χ*^*2*^	*P value*
	Con-ICC (%)	HBV-ICC (%)	Stone-ICC (%)		
**Sex**					
Male	127(48.7)	63(61.8)	33(38.8)	10.073	0.006
Female	134(51.3)	39(38.2)	52(61.2)		
**Age (year)**					
≤ 55	110(42.1)	51(50.0)	26(30.6)	7.226	0.027
> 55	151(57.9)	51(50.0)	59(69.4		
**Obstructive jaundice**					
No	246(94.3)	100(98.0)	69(81.2)	21.723	< 0.001
Yes	15(5.7)	2(2.0)	16(18.8)		
AFP (ng/ml)					
≤ 7.0	203(78.8)	77(75.5)	63(74.1)	0.563	0.755
> 7.0	58(22.2)	25(24.5)	22(25.9)		
CEA (ng/ml)					
≤ 5.0	200(76.6)	77(75.5)	60(70.6)	1.260	0.533
> 5.0	61(23.4)	25(24.5)	25(29.4)		
**CA19-9(U/ml)**					
≤ 39.0	121(46.1)	48(47.1)	25(29.4)	8.260	0.016
> 39.0	140(53.6)	54(52.9)	60(70.6)		
CA125(U/ml)					
≤ 35.0	161(61.7)	59(57.8)	51(60.0)	0.464	0.793
> 35.0	100(38.3)	43(42.2)	34(40.0)		
**Child–Pugh Grade**					
Grade A	247(94.6)	99(97.1)	74(87.1)	8.750	0.013
Grade B	14(5.4)	3(2.9)	11(12.9)		
Type of resection					
Wedge resection	108(41.4)	49(48.0)	28(32.9)	8.722	0.068
Minor hepatectomy	109(41.8)	43(42.2)	36(42.4)		
Major hepatectomy	44(16.9)	10(9.8)	21(24.7)		
Tumor differentiation					
Well	17(6.5)	4(3.9)	7(8.2)	3.227	0.521
Moderate	164(62.8)	59(57.8)	49(57.6)		
Poor	80(30.7)	39(38.2)	29(34.1)		
**Tumor location**					
Left	128(49.0)	36(35.3)	56(65.9)	18.423	0.001
Right	99(37.9)	53(52.0)	23(27.1)		
Left and right	34(13.0)	13(12.7)	6(7.1)		
**Morphologic grape**					
Mass-forming	221(84.7)	88(86.3)	51(60.0)	28.811	< 0.001
Periductal infiltrating	24(9.2)	8(7.8)	17(20.0)		
Intraductal growth	16(6.1)	6(5.9)	17(20.0)		
Tumor size (cm)					
≤ 5.0	135(51.7)	52(51.0)	50(58.8)	1.493	0.474
> 5.0	126(48.3)	50(49.0)	35(41.2)		
Major vascular invasion					
No	217(83.1)	91(89.2)	69(81.2)	2.725	0.256
Yes	44(16.9)	11(10.8)	16(18.8)		
Microvascular invasion					
No	235(90.0)	87(85.3)	71(83.5)	3.246	0.197
Yes	26(10.0)	15(14.7)	14(16.5)		
**Perineural invasion**					
No	223(85.4)	96(94.1)	66(77.6)	10.535	0.005
Yes	38(14.6)	6(5.9)	19(22.4)		
**Liver capsule involvement**					
No	180(69.0)	64(62.7)	69(81.2)	7.722	0.021
Yes	81(31.0)	38(37.3)	16(18.8)		
AJCC 8th edition T stage					
T_1a_/T_1b_	63(24.1)	27(26.5)	26(30.6)	3.817	0.431
T_2_	132(50.6)	45(44.1)	43(50.6)		
T_3_/T_4_	66(25.3)	30(29.4)	16(18.8)		
**AJCC 8th edition N stage**					
N0	193(73.9)	82(80.4)	61(71.8)	2.211	0.031
N1	68(26.1)	20(19.6)	24(28.2)		
AJCC 8th edition TNM stage					
IA/IB	119(45.6)	42(41.2)	45(52.9)	3.540	0.472
II	40(15.3)	20(19.6)	10(11.8)		
IIIA/IIIB/IV	102(39.1)	40(39.2)	30(35.3)		

### Survival analysis on OS and RFS in the whole cohort

The 1-, 3-, and 5-year OS rates of ICC patients were 84.9%, 42.4%, and 20.0%, and 1-, 3-, and 5-year RFS rates of ICC patients were 56.5%, 20.6%, and 10.3%, respectively. Median OS and RFS were 28.0 and 14.9 months, respectively. Univariate analysis showed that different etiology was a prognostic factor for OS and RFS of patients with ICC after radical resection, respectively (Supplemental Fig. 1, *P* < 0.05). Multivariate analysis showed that different etiology was an independent risk factor for OS. Detailed results of the univariate and multivariate analysis are shown in Table 2.

**Fig. 1:**
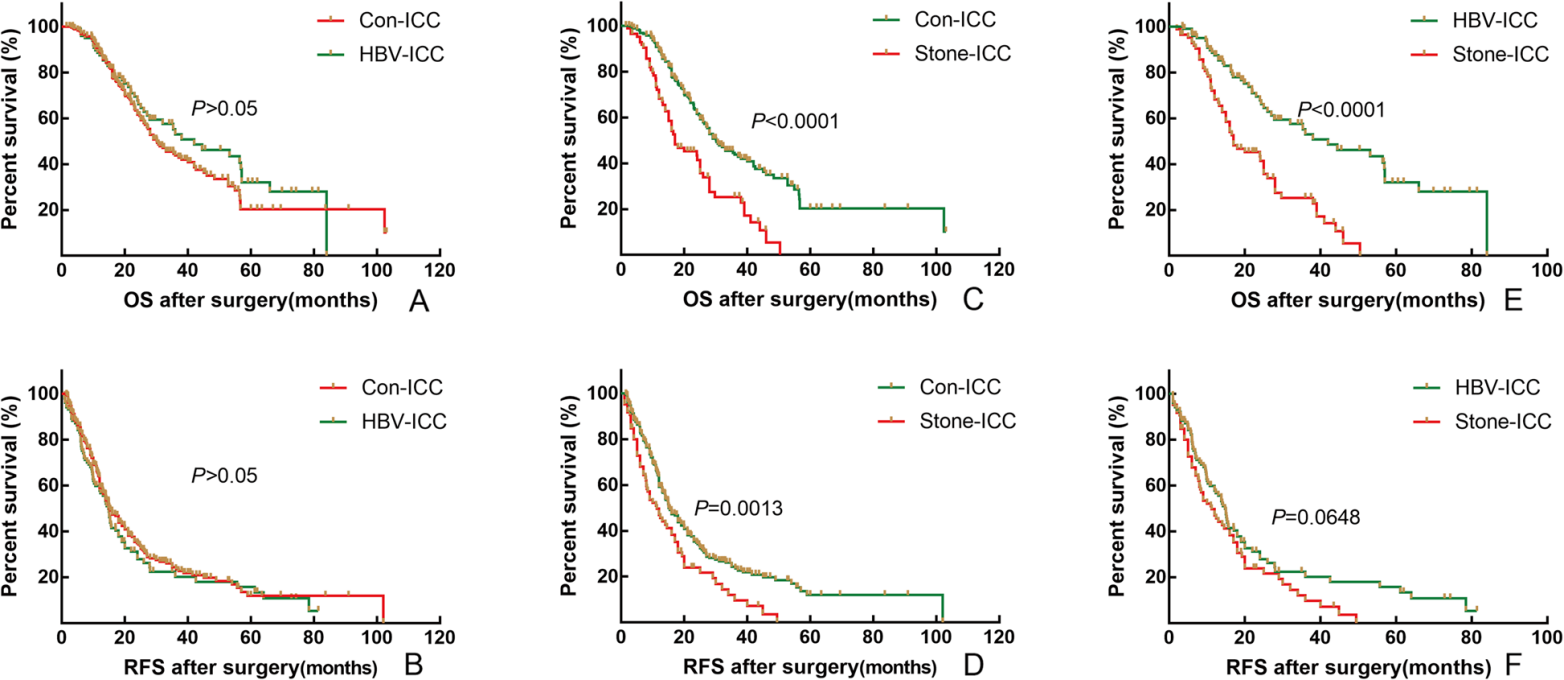
Comparison of overall survival and relapse-free survival after radical resection between conventional ICC and HBV-ICC (**A** and **B**), between conventional ICC and Stone-ICC (**C** and **D**), and between HBV-ICC and Stone-ICC (**E** and **F**) before PSM

**Table 2:** Univariate and multivariate analysis of prognosis for ICC after radical resection

	OS				RFS			
	Univariate analysis		Multivariate analysis		Univariate analysis		Multivariate analysis	
	*HR (*95%*CI)*	*P*	*HR (*95%*CI)*	*P*	*HR (*95%*CI)*	*P*	*HR (*95%*CI)*	*P*
Sex								
Female *vs* Male	0.935(0.721 ~ 1.212)	0.610			0.891(0.717 ~ 1.109)	0.302		
Age (year)								
> 55 *vs* ≤ 55	1.242(0.955 ~ 1.615)	0.105			1.046(0.838 ~ 1.305)	0.693		
Obstructive jaundice								
Yes *vs* No	1.172(0.872 ~ 2.150)	0.175			1.090(0.729 ~ 1.628)	0.675		
Etiology								
HBV-ICC *vs* Con-ICC	0.801(0.567 ~ 1.130)	0.206	0.862(0.608 ~ 1.223)	0.405	1.143(0.873 ~ 1.498)	0.331		
Stone-ICC *vs* Con-ICC	2.185(1.597 ~ 2.989)	< 0.001	2.009(1.447 ~ 2.789)	< 0.001	1.563(1.181 ~ 2.068)	0.002		
AFP (ng/ml)								
> 7.0 *vs* ≤ 7.0	1.137(0.834 ~ 1.550)	0.416			1.041(0.804 ~ 1.348)	0.761		
CEA (ng/ml)								
> 5.0 *vs* ≤ 5.0	1.439(1.076 ~ 1.923)	0.014			1.348(1.054 ~ 1.725)	0.017		
CA19-9(U/ml)								
> 39.0 *vs* ≤ 39.0	1.378(1.056 ~ 1.798)	0.018			1.370(1.097 ~ 1.711)	0.006	1.252(1.010 ~ 1.568)	0.030
CA125(U/ml)								
> 35.0 *vs* ≤ 35.0	1.604(1.218 ~ 2.111)	0.001			1.308(1.046 ~ 1.635)	0.019		
Child–Pugh Grade								
Grade B *vs* A	1.150(0.709 ~ 1.866)	0.570			1.006(0.662 ~ 1.526)	0.979		
Type of resection								
Minor hepatectomy *vs* Wedge resection	1.718(1.284 ~ 2.297)	< 0.001	1.157(1.127 ~ 2.043)	0.006	1.684(1.318 ~ 2.151)	< 0.001	1.515(1.183 ~ 1.941)	0.001
Major hepatectomy *vs* Wedge resection	1.962(1.344 ~ 2.864)	< 0.001	1.405(1.149 ~ 2.080)	0.019	1.829(1.324 ~ 2.528)	< 0.001	1.733(1.245 ~ 2.411)	0.001
Lymphadenectomy								
Yes *vs* No	0.909(0.697 ~ 1.185)	0.479			1.048(0.837 ~ 1.312)	0.683		
Tumor differentiation								
Moderate *vs* Well	1.318(0.739 ~ 2.352)	0.350			1.002(0.644 ~ 1.560)	0.993		
Poor *vs* Well	2.243(1.228 ~ 4.095)	0.009			1.597(1.108 ~ 2.302)	0.012		
Tumor location								
Right *vs* Left	0.689(0.518 ~ 1.129)	0.069			0.890(0.704 ~ 1.127)	0.334		
Left and right *vs* Left	0.857(0.556 ~ 1.321)	0.485			0.976(0.685 ~ 1.391)	0.895		
Morphologic grape								
Periductal infiltrating *vs* Mass-forming	0.871(0.559 ~ 1.358)	0.543			0.997(0.700 ~ 1.420)	0.986		
Intraductal growth *vs* Mass-forming	0.825(0.502 ~ 1.357)	0.448			0.789(0.530 ~ 1.174)	0.789		
Tumor size (cm)								
> 5.0 *vs* ≤ 5.0	1.517(1.148 ~ 2.003)	0.003	1.352(1.034 ~ 1.797)	0.028	1.164(0.936 ~ 1.149)	0.173		
Major vascular invasion								
Yes *vs* No	1.716(1.249 ~ 2.357)	0.001			1.606(1.217 ~ 2.121)	0.001		
Microvascular invasion								
Yes *vs* No	1.506(1.009 ~ 2.248)	0.045			1.748(1.388 ~ 2.202)	< 0.001	1.788(1.400 ~ 2.257)	< 0.001
Perineural invasion								
Yes *vs* No	2.068(1.421 ~ 3.008)	< 0.001			1.331(0.964 ~ 1.838)	0.083		
Liver capsule involvement								
Yes *vs* No	1.136(0.857 ~ 1.507)	0.375			1.262(0.894 ~ 1.783)	0.186		
AJCC 8th edition T stage								
T_2_ *vs* T_1a_/T_1b_	1.317(0.947 ~ 1.831)	0.101			1.164(0.885 ~ 1.532)	0.277		
T_3_/T_4_ *vs* T_1a_/T_1b_	1.559(1.080 ~ 2.250)	0.018			1.713(1.273 ~ 2.305)	< 0.001		
AJCC 8th edition N stage								
N1 *vs* N0	1.949(1.459 ~ 2.603)	< 0.001	1.791(1.334 ~ 2.406)	< 0.001	1.648(1.289 ~ 2.106)	< 0.001	1.378(1.070 ~ 1.776)	0.013
AJCC 8th edition TNM stage								
II *vs* IA/IB	1.362(0.947 ~ 1.961)	0.096			1.258(0.907 ~ 1.746)	0.170		
IIIA/IIIB/IV *vs* IA/IB	1.521(1.138 ~ 2.033)	0.005			1.732(1.365 ~ 2.198)	< 0.001		
Adjuvant chemotherapy								
Yes *vs* No	0.685(0.520 ~ 0.902)	0.007	0.632(0.469 ~ 0.851)	0.003	0.715(0.568 ~ 0.901)	0.005	0.655(0.517 ~ 0.831)	< 0.001

**Fig. 2:**
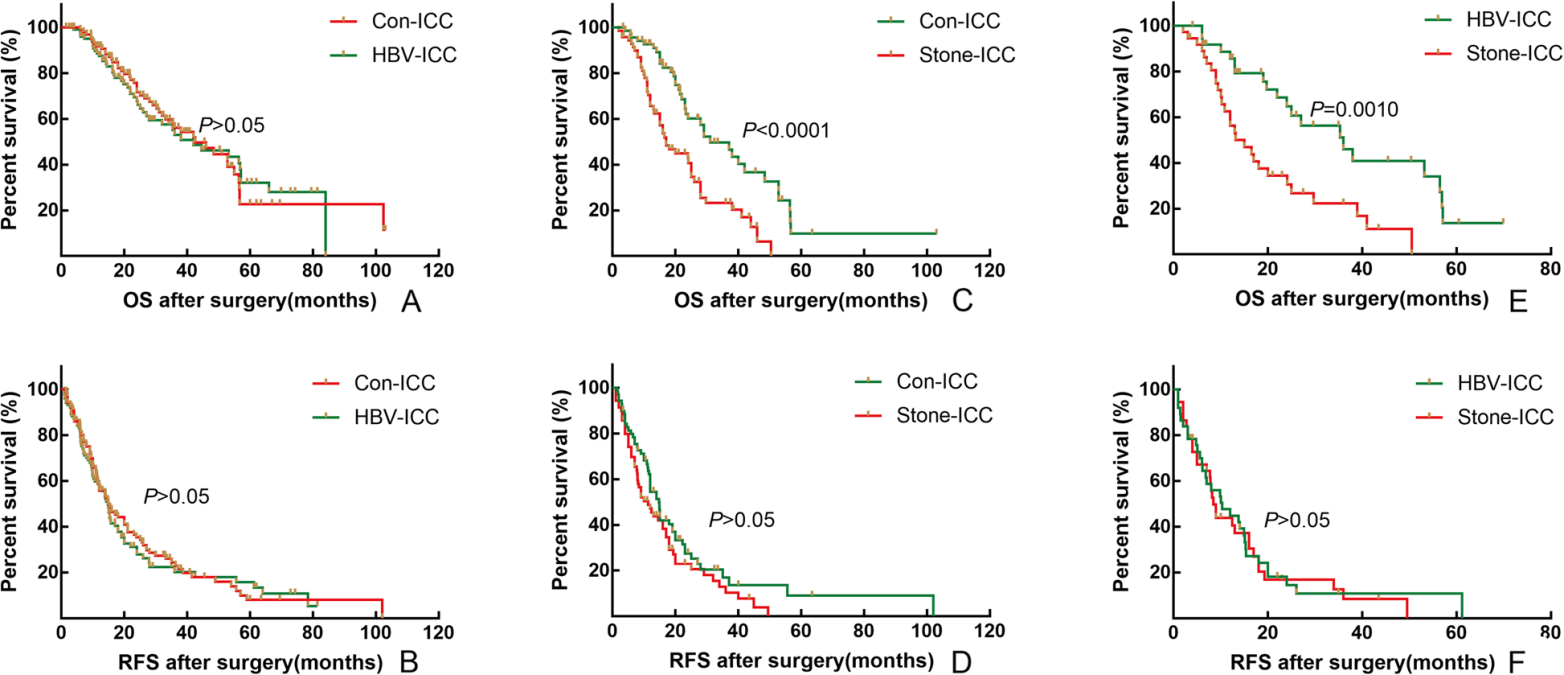
Comparison of overall survival and relapse-free survival after radical resection between conventional ICC and HBV-ICC (**A** and **B**), between conventional ICC and Stone-ICC (**C** and **D**), and between HBV-ICC and Stone-ICC (**E** and **F**) after PSM

To eliminate the differences and be comparable of baseline clinicopathological characteristics between related two subtypes, a 1:1 PSM was utilized to identify 102 pairs of patients with Con-ICC and HBV-ICC, 70 pairs of patients with Con-ICC and Stone-ICC, 37 pairs of patients with HBV-ICC and Stone-ICC. The baseline characteristics of three groups before and after PSM are showed in Supplemental Tables 1–3. Before and after PSM, the OS and RFS of patients with Con-ICC and HBV-ICC were not statistically significant (Fig. 1, 2-A and B, *P* > 0.05); however, the OS of patients with Stone-ICC was worse than those of Con-ICC and HBV-ICC subtypes (Fig. 1, 2-C and E, *P* < 0.05), while the RFS of patients with Stone-ICC was equivalent to patients with Con-ICC and HBV-ICC (Fig. 2- D and F, *P* > 0.05). Therefore, the results showed that the prognosis of Stone-ICC subtype was significantly worse than those of Con-ICC and HBV-ICC subtypes.

### Comparison of recurrence and survival

**Fig. 3:**
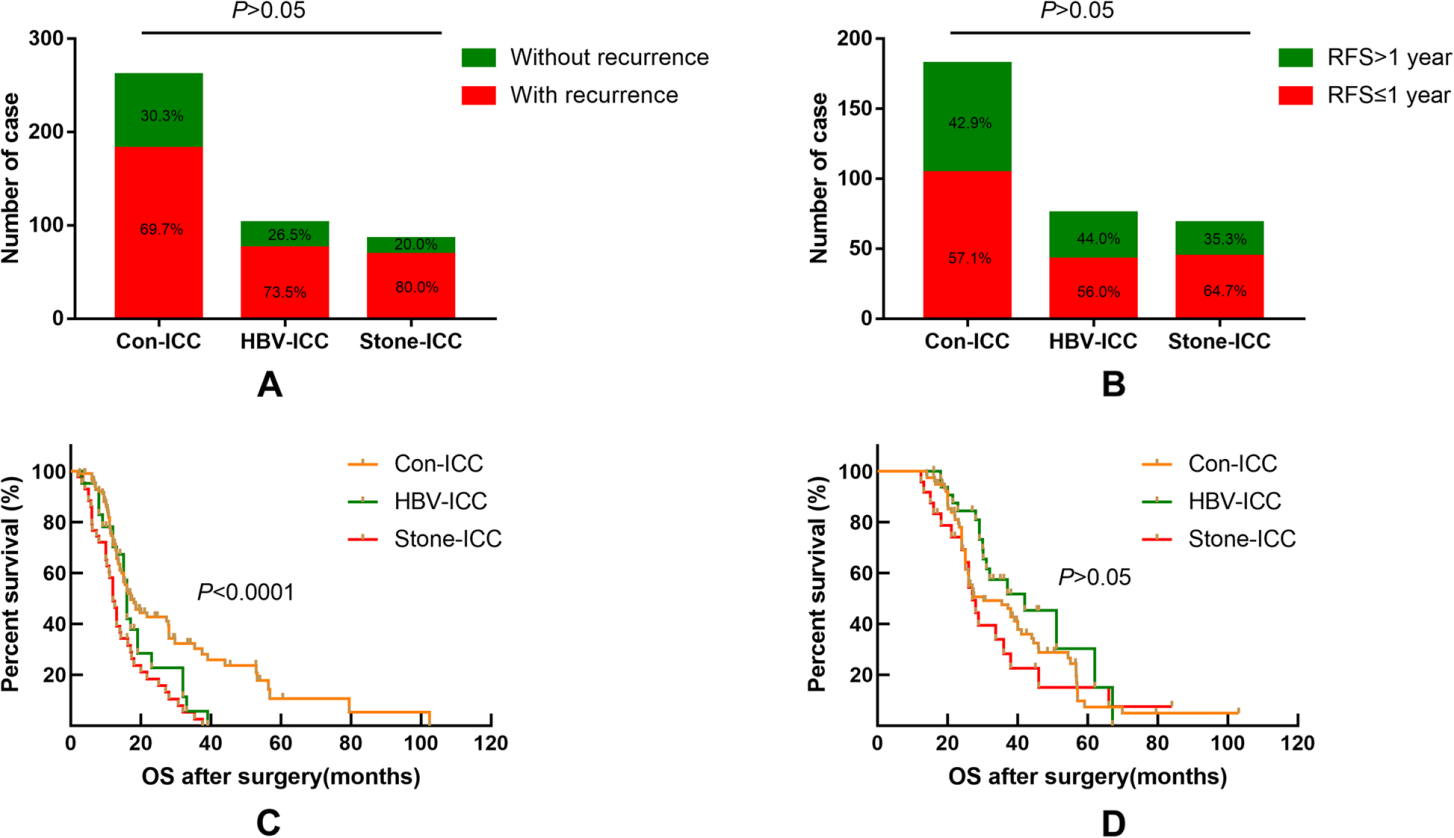
Comparison of overall recurrence ratio (**A**), recurrence time ≤ 1 year and > 1 year (**B**), and overall survival time with recurrence time ≤ 1 year (**C**) and overall survival time with recurrence time > 1 year after radical resection for conventional ICC, HBV-ICC and Stone-ICC

By comparing overall recurrence, early recurrence (RFS ≤ 1 year after surgery,), and OS for the different etiological subtypes in a proportion of ICC patients, the results showed that there was no statistical difference in the proportion of patients with overall recurrence and early recurrence (Fig. 3-A and B, *P* > 0.05). By further comparing the difference of OS in early recurrence and non-early recurrence patients, there was a statistical difference with the three etiological subtypes in early recurrence patients, and Stone-ICC tended to have a worse prognosis (Fig. 3-C, *P* < 0.05), while there was no statistical difference in non-early recurrence patients (Fig. 3-D, *P* > 0.05). Therefore, the survival difference of the three etiological subtypes was mainly for OS in patients with early recurrence.

### Comparison of OS and RFS in ACT

**Fig. 4:**
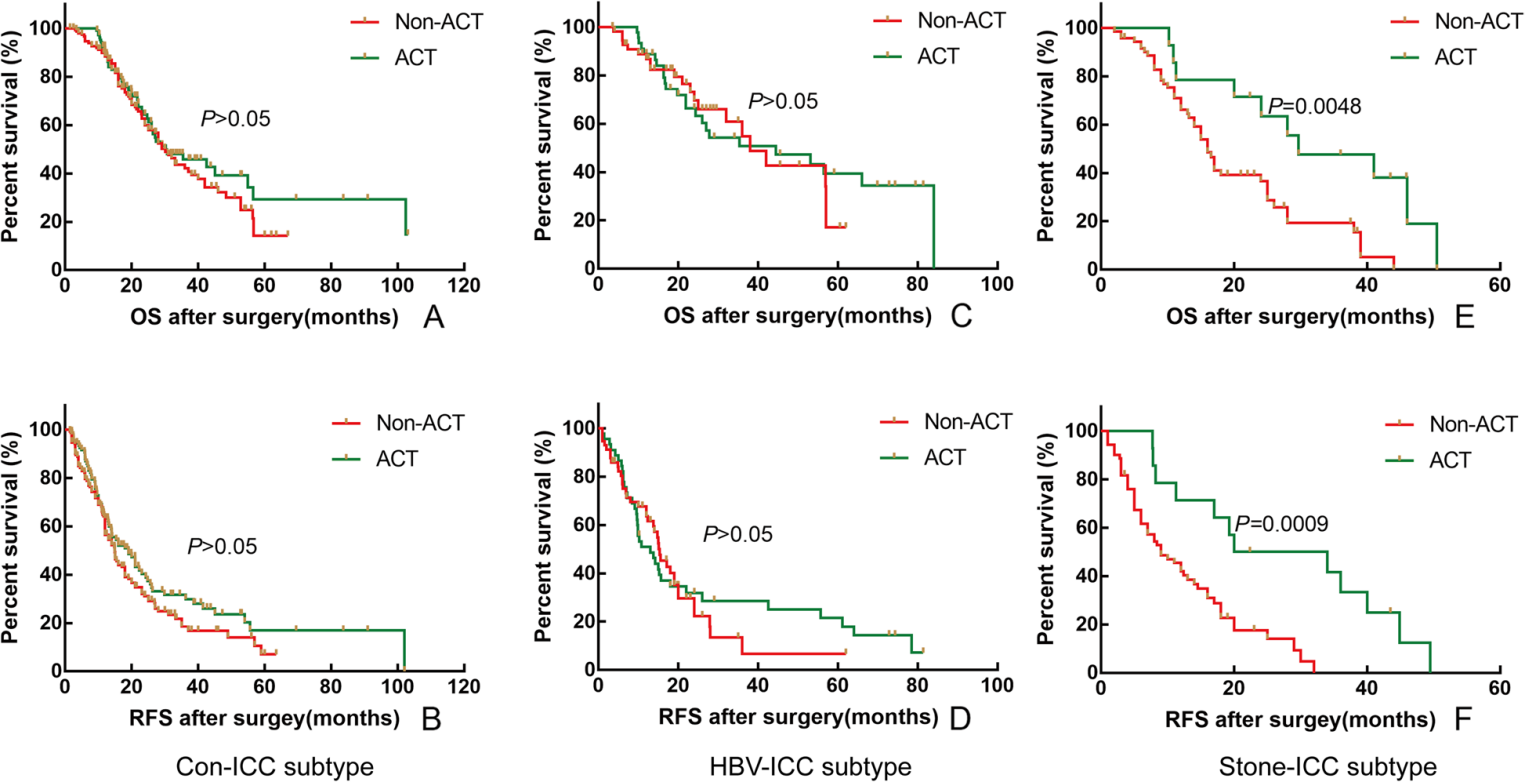
Comparison of overall survival and relapse-free survival in adjuvant chemotherapy after radical resection between conventional ICC and HBV-ICC (**A** and **B**), between conventional ICC and Stone-ICC (**C** and **D**), and between HBV-ICC and Stone-ICC (**E** and **F**)

To determine whether the ACT regimens affected the prognosis of patients, we first analyzed the prognosis differences among the four regimens for patients with postoperative ACT, and the results showed that there was no difference in OS and RFS among different chemotherapy regimens (*P* > 0.05). By analyzing the prognostic improvement value of ACT for different etiological subtypes in ICC patients, the results showed that in Con-ICC patients, the median OS was 30.2 months and 30.2 months, and the median RFS was 14.9 months and 19.0 months for non-ACT and ACT patients, respectively (Fig. 4-A and B, *P* > 0.05); in HBV-ICC patients, the median OS was 38.0 months and 44.5 months, and the median RFS was 13.0 months and 15.3 months for non-ACT and ACT patients, respectively (Fig. 4-C and D, *P* > 0.05); in Stone-ICC patients, the median OS was 16.0 months and 29.7 months, and the median RFS was 9.0 months and 20.0 months for non-ACT and ACT patients, respectively (Fig. 4-E and F, *P* < 0.05). Therefore, postoperative ACT can improve the OS and RFS of Stone-ICC patients effectively.

## Discussion

Hepatolithiasis and HBV infection as the two most common risk factors for ICC in China, and ICC without a clear cause (conventional-ICC) were the mainly specific pathogenic factors in present studies [14–17, 20]. Therefore, we divided the etiology of ICC patients into three subtypes, Con-ICC (no identifiable cause), HBV-ICC, and Stone-ICC. Survival analysis showed that the OS and RFS were statistically significant among the three etiological subtypes, and the etiology of ICC was identified as an independent risk factor for OS. To further compare the survival differences between related two etiological subtypes, PSM was conducted to eliminate differences in baseline clinicopathological characteristics. After PSM, the OS of patients with Stone-ICC was worse than those of Con-ICC and HBV-ICC subtypes, while the RFS of patients with Stone-ICC was equivalent to patients with Con-ICC and HBV-ICC. Wang et al. [21] conducted a single institutional study and found that Stone-ICC had a worse prognosis compared to HBV-ICC after PSM. Similarly, Zhang et al. [20] conducted an international multi-institutional study and also found that Stone-ICC had a worse prognosis than Con-ICC and HBV-ICC subtypes, and they further found that Stone-ICC and Con-ICC had statistical differences in OS and RFS, while Stone-ICC and HBV-ICC had no difference on prognosis after PSM. In this study, we also found that there was no statistical difference in the proportion of patients with overall recurrence, early recurrence and OS in non-early recurrence patients with the three etiological subtypes, while there was a statistical difference of OS ≤ 1 year, OS with 1 ~ 3 year and OS > 3 years in early recurrence patients. So, the survival difference of ICC with the different etiological subtypes was mainly in OS, especially in patients with early recurrence.

To further explore the reasons for the differences in the prognosis of ICC with different etiology, we analyzed the differences in clinicopathological characteristics of ICC with the three etiological subtypes. Based on the above results, we considered that the prognostic differences of ICC with different etiology had a strong correlation with its clinicopathological characteristics. Widespread epidemics with HBV infection in China increase integration of HBV gene fragments into liver cells, which contributes to ICC and also causes the HBV-ICC of China with highest distribution in the world [22–24]. In this study, the results showed that the prognosis of patients with HBV-ICC was better than those of Con-ICC and Stone-ICC subtypes. Ding et al. [25] have found that the patients with HBV-ICC can activate the immune memory produced by HBV infection previously, thereby enhancing anti-tumor immunity, and Iida et al. [26] have also revealed that HBV-ICC conferred a low risk of lymph node metastasis for postoperative recurrence, which may be the reason that HBV-ICC has a relatively better prognosis than the other two etiological subtypes. Many studies [21, 27, 28] also found that HBV infection was a favorable prognostic factor for ICC after surgery, which was consistent with our results.

Unfortunately, patients with Stone-ICC had the worst prognosis in the study. Wang et al.[21] revealed that patients with Stone-ICC were often difficult to differentiate with benign biliary strictures, resulting in patients who were mostly diagnosed in the advanced stage. Due to the presence of hepatolithiasis, it can lead to long-term chronic inflammation, followed by dysplasia and multiple tumors. In addition, the proportion of Stone-ICC with elevated CA19-9, multiple tumors, and lymph node metastasis was significantly higher than the other two etiological types [21, 29, 30]. In this study, Stone-ICC had a higher proportion of CA19-9 > 39.0 U/ml, presence of perineural invasion, morphologic grape with periductal infiltrating and intraductal growth, and N1 stage compared with HBV-ICC and Con-ICC, which could explain the reason why Stone-ICC had a poor prognosis. Thus, we suggest a more aggressive preventive surgery for those patients with a long-term history of hepatolithiasis to avoid the presence of ICC.

At present, due to limited prospective data on the benefits of systemic ACT for ICC patients after radical resection [9], whether postoperative ACT could improve the prognosis of ICC patients is still controversial [31, 32]. In this study, we found that postoperative ACT could improve the OS and RFS effectively, and non-ACT was identified as an independent risk factor for ICC patients (Table 2). Recently, some studies [33, 34] proved that postoperative ACT was beneficial to the prognosis for ICC after surgery. By analyzing the prognostic improvement value of ACT for different etiological subtypes in ICC patients, the results showed that postoperative ACT was a benefit to Stone-ICC patients on OS and RFS. However, ACT did not significantly improve the prognosis for Con-ICC and HBV-ICC patients. Altman et al. [35] and Ke et al. [36] reported that ACT improving OS was related to more patients with lymph node-positive or T3/T4 stage. We considered that ACT improving the prognosis of Stone-ICC was related to its high proportion of elevated CA19-9, perineural invasion, N1 stage patients. Of course, whether ACT can improve the prognosis of Con-ICC and HBV-ICC patients still needs further research.

However, there were several limitations in our study. The study included 448 ICC patients after radical resection from 10 medical centers in China, which effectively increased the universality of the study, while the sample size was still relatively small. Besides, the study did not include ICC patients from Western countries, because the role of hepatolithiasis and HBV infection was not as important as those patients in China. In addition, the preoperative inflammatory indicators were not available, so the differences of the three etiological subtypes of inflammatory response were not further compared. Accordingly, we should clarify the molecular mechanisms and prognostic monitoring indicators for ICC with different etiology in the future, so as to provide references and decision support for the individualized diagnosis, treatment and prevention for ICC patients.

## Conclusion

In conclusion, this study retrospectively analyzed 448 ICC patients with different etiology after radical resection, the survival difference of ICC with different etiological subtypes was mainly in OS, especially in patients with early recurrence, but there was no difference in RFS. Importantly, different etiology was identified as an independent risk factor for OS in patients with ICC after radical resection. The prognosis of Stone-ICC patients was significantly worse than those of Con-ICC and HBV-ICC patients. Interestingly, postoperative ACT can improve the prognosis of Stone-ICC patients.

## Supplementary Information

**Additional file 1.** 

**Additional file 2:** **Supplement Fig 1.** Comparison of overall survival and relapse-free survival after radical resection for conventional ICC, HBV-ICC and Stone-ICC (A and B).

## Data Availability

This manuscript contains all associated data.

## Abbreviations

ICC: Intrahepatic cholangiocarcinoma
ACT: Adjuvant chemotherapy
PSM: Propensity score matching
PSC: Primary sclerosing cholangitis
NLFLD: Non-alcoholic fatty liver disease
HBV: Hepatitis B virus
OS: Overall survival
RFS: Relapse-free survival
