# Supplementary material for: Recurrence and prognosis in intrahepatic cholangiocarcinoma patients with different etiology after radical resection: a multi-institutional study
Source: BMC Cancer. 2022 Mar 26;22:329. doi: 10.1186/s12885-022-09448-w (PMC8962079; doi:10.1186/s12885-022-09448-w)
Supplement: Supplementary file 1 — Additional file 1. [file 12885_2022_9448_MOESM1_ESM.docx]

Supplemental Table 1. Demographics and clinical characteristics of patients with Con-ICC and HBV-ICC before and after PSM

|  | Before PSM | | χ^2^ | *P* | After PSM | | χ^2^ | *P* |
| --- | --- | --- | --- | --- | --- | --- | --- | --- |
|  | Con-ICC (%) | HBV -ICC (%) |  |  | Con-ICC (%) | HBV -ICC (%) |  |  |
| **Sex** |  |  |  |  |  |  |  |  |
| Male | 127(48.7) | 63(61.8) | 5.050 | 0.025 | 62(60.8) | 63(61.8) | 0.021 | 0.886 |
| Female | 134(51.3) | 39(38.2) |  |  | 40(39.2) | 39(38.2) |  |  |
| Age (year) |  |  |  |  |  |  |  |  |
| ≤55 | 110(42.1) | 51(50.0) | 1.833 | 0.176 | 42(41.2) | 51(50.0) | 1.601 | 0.206 |
| >55 | 151(57.9) | 51(50.0) |  |  | 60(54.0) | 51(50.0) |  |  |
| Obstructive jaundice |  |  |  |  |  |  |  |  |
| No | 246(94.3) | 100(98.0) | 2.355 | 0.125 | 99(97.1) | 100(98.0) | 0.205 | 0.651 |
| Yes | 15(5.7) | 2(2.0) |  |  | 3(2.9) | 2(2.0) |  |  |
| AFP (ng/ml) |  |  |  |  |  |  |  |  |
| ≤7.0 | 203(78.8) | 77(75.5) | 0.218 | 0.641 | 81(79.4) | 77(75.5) | 0.449 | 0.503 |
| >7.0 | 58(22.2) | 25(24.5) |  |  | 21(20.6) | 25(24.5) |  |  |
| CEA (ng/ml) |  |  |  |  |  |  |  |  |
| ≤5.0 | 200(76.6) | 77(75.5) | 0.053 | 0.819 | 76(74.5) | 77(75.5) | 0.026 | 0.872 |
| >5.0 | 61(23.4) | 25(24.5) |  |  | 26(25.5) | 25(24.5) |  |  |
| CA19-9(U/ml) |  |  |  |  |  |  |  |  |
| ≤39.0 | 121(46.1) | 48(47.1) | 0.014 | 0.905 | 45(44.1) | 48(47.1) | 0.178 | 0.673 |
| >39.0 | 140(53.6) | 54(52.9) |  |  | 57(55.9) | 54(52.9) |  |  |
| CA125(U/ml) |  |  |  |  |  |  |  |  |
| ≤35.0 | 161(61.7) | 59(57.8) | 0.454 | 0.501 | 68(66.7) | 59(57.8) | 1.690 | 0.194 |
| >35.0 | 100(38.3) | 43(42.2) |  |  | 34(34.3) | 43(42.2) |  |  |
| Child-Pugh Grade |  |  |  |  |  |  |  |  |
| Grade A | 247(94.6) | 99(97.1) | 0.964 | 0.326 | 96(94.1) | 99(97.1) | 1.046 | 0.306 |
| Grade B | 14(5.4) | 3(2.9) |  |  | 6(5.9) | 3(2.9) |  |  |
| Type of resection |  |  |  |  |  |  |  |  |
| Wedge resection | 108(41.4) | 49(48.0) | 3.208 | 0.201 | 52(51.0) | 49(48.0) | 0.580 | 0.748 |
| Minor hepatectomy | 109(41.8) | 43(42.2) |  |  | 38(37.3) | 43(42.2) |  |  |
| Major hepatectomy | 44(16.9) | 10(9.8) |  |  | 12(11.8) | 10(9.8) |  |  |
| Tumor differentiation |  |  |  |  |  |  |  |  |
| Well | 17(6.5) | 4(3.9) | 2.436 | 0.296 | 6(5.9) | 4(3.9) | 0.817 | 0.665 |
| Moderate | 164(62.8) | 59(57.8) |  |  | 62(60.8) | 59(57.8) |  |  |
| Poor | 80(30.7) | 39(38.2) |  |  | 34(33.3) | 39(38.2) |  |  |
| **Tumor location** |  |  |  |  |  |  |  |  |
| Left | 128(49.0) | 36(35.3) | 6.520 | 0.038 | 35(34.3) | 36(35.3) | 0.166 | 0.920 |
| Right | 99(37.9) | 53(52.0) |  |  | 52(51.0) | 53(52.0) |  |  |
| Left and right | 34(13.0) | 13(12.7) |  |  | 15(14.7) | 13(12.7) |  |  |
| Morphologic grape |  |  |  |  |  |  |  |  |
| Mass-forming | 221(84.7) | 88(86.3) | 0.182 | 0.913 | 85(83.3) | 88(86.3) | 0.351 | 0.839 |
| Periductal infiltrating | 24(9.2) | 8(7.8) |  |  | 10(9.8) | 8(7.8) |  |  |
| Intraductal growth | 16(6.1) | 6(5.9) |  |  | 7(6.9) | 6(5.9) |  |  |
| Tumor size (cm) |  |  |  |  |  |  |  |  |
| ≤5.0 | 135(51.7) | 52(51.0) | 0.016 | 0.899 | 50(49.0) | 52(51.0) | 0.078 | 0.779 |
| >5.0 | 126(48.3) | 50(49.0) |  |  | 52(51.0) | 50(49.0) |  |  |
| Major vascular invasion |  |  |  |  |  |  |  |  |
| No | 217(83.1) | 91(89.2) | 2.105 | 0.147 | 87(85.3) | 91(89.2) | 0.705 | 0.401 |
| Yes | 44(16.9) | 11(10.8) |  |  | 15(14.7) | 11(10.8) |  |  |
| Microvascular invasion |  |  |  |  |  |  |  |  |
| No | 235(90.0) | 87(85.3) | 1.648 | 0.199 | 94(92.2) | 87(85.3) | 2.401 | 0.121 |
| Yes | 26(10.0) | 15(14.7) |  |  | 8(7.8) | 15(14.7) |  |  |
| **Perineural invasion** |  |  |  |  |  |  |  |  |
| No | 223(85.4) | 96(94.1) | 5.184 | 0.023 | 99(97.1) | 96(94.1) | 1.046 | 0.306 |
| Yes | 38(14.6) | 6(5.9) |  |  | 3(2.9) | 6(5.9) |  |  |
| Liver capsule involvement |  |  |  |  |  |  |  |  |
| No | 180(69.0) | 64(62.7) | 1.288 | 0.256 | 64(62.7) | 64(62.7) | 0.000 | 1.000 |
| Yes | 81(31.0) | 38(37.3) |  |  | 38(37.3) | 38(37.3) |  |  |
| AJCC 8th edition T stage |  |  |  |  |  |  |  |  |
| T_1a_/T_1b_ | 63(24.1) | 27(26.5) | 1.260 | 0.533 | 25(24.5) | 27(26.5) | 0.738 | 0.692 |
| T_2_ | 132(50.6) | 45(44.1) |  |  | 51(50.0) | 45(44.1) |  |  |
| T_3_/T_4_ | 66(25.3) | 30(29.4) |  |  | 26(25.5) | 30(29.4) |  |  |
| AJCC 8th edition N stage |  |  |  |  |  |  |  |  |
| N0 | 193(73.9) | 82(80.4) | 1.659 | 0.198 | 83(81.4) | 82(80.4) | 0.032 | 0.859 |
| N1 | 68(26.1) | 20(19.6) |  |  | 19(18.6) | 20(19.6) |  |  |
| AJCC 8th edition TNM stage |  |  |  |  |  |  |  |  |
| IA/IB | 119(45.6) | 42(41.2) | 1.137 | 0.566 | 45(44.1) | 42(41.2) | 0.347 | 0.841 |
| II | 40(15.3) | 20(19.6) |  |  | 17(16.7) | 20(19.6) |  |  |
| IIIA/IIIB/IV | 102(39.1) | 40(39.2) |  |  | 40(39.2) | 40(39.2) |  |  |

Supplemental Table 2. Demographics and clinical characteristics of patients with Con-ICC and Stone-ICC before and after PSM

|  | Before PSM | | χ^2^ | *P* | After PSM | | χ^2^ | *P* |
| --- | --- | --- | --- | --- | --- | --- | --- | --- |
|  | Con-ICC (%) | Stone-ICC (%) |  |  | Con-ICC (%) | Stone-ICC (%) |  |  |
| Sex |  |  |  |  |  |  |  |  |
| Male | 127(48.7) | 33(38.8) | 2.495 | 0.114 | 30(42.9) | 29(41.4) | 0.029 | 0.864 |
| Female | 134(51.3) | 52(61.2) |  |  | 40(57.1) | 41(58.9) |  |  |
| Age (year) |  |  |  |  |  |  |  |  |
| ≤55 | 110(42.1) | 26(30.6) | 3.590 | 0.058 | 23(32.9) | 18(25.7) | 0.862 | 0.353 |
| >55 | 151(57.9) | 59(69.4) |  |  | 47(67.1) | 52(74.3) |  |  |
| **Obstructive jaundice** |  |  |  |  |  |  |  |  |
| No | 246(94.3) | 69(81.2) | 13.441 | <0.001 | 61(87.1) | 59(84.3) | 0.233 | 0.629 |
| Yes | 15(5.7) | 16(18.8) |  |  | 9(12.9) | 11(15.7) |  |  |
| AFP (ng/ml) |  |  |  |  |  |  |  |  |
| ≤7.0 | 203(78.8) | 63(74.1) | 0.483 | 0.487 | 48(68.6) | 50(71.4) | 0.136 | 0.712 |
| >7.0 | 58(22.2) | 22(25.9) |  |  | 22(31.4) | 20(28.6) |  |  |
| CEA (ng/ml) |  |  |  |  |  |  |  |  |
| ≤5.0 | 200(76.6) | 60(70.6) | 1.252 | 0.263 | 47(67.1) | 48(68.6) | 0.033 | 0.856 |
| >5.0 | 61(23.4) | 25(29.4) |  |  | 23(32.9) | 22(31.4) |  |  |
| **CA19-9(U/ml)** |  |  |  |  |  |  |  |  |
| ≤39.0 | 121(46.1) | 25(29.4) | 7.551 | 0.006 | 18(25.7) | 21(30.0) | 0.320 | 0.572 |
| >39.0 | 140(53.6) | 60(70.6) |  |  | 52(74.3) | 49(70.0) |  |  |
| CA125(U/ml) |  |  |  |  |  |  |  |  |
| ≤35.0 | 161(61.7) | 51(60.0) | 0.077 | 0.782 | 46(65.7) | 43(61.4) | 0.278 | 0.598 |
| >35.0 | 100(38.3) | 34(40.0) |  |  | 24(34.3) | 27(38.6) |  |  |
| **Child-Pugh Grade** |  |  |  |  |  |  |  |  |
| Grade A | 247(94.6) | 74(87.1) | 5.492 | 0.019 | 64(91.4) | 61(87.1) | 0.672 | 0.412 |
| Grade B | 14(5.4) | 11(12.9) |  |  | 6(8.6) | 9(12.9) |  |  |
| Type of resection |  |  |  |  |  |  |  |  |
| Wedge resection | 108(41.4) | 28(32.9) | 3.269 | 0.195 | 25(35.7) | 23(32.9) | 2.797 | 0.247 |
| Minor hepatectomy | 109(41.8) | 36(42.4) |  |  | 34(48.4) | 28(40.0) |  |  |
| Major hepatectomy | 44(16.9) | 21(24.7) |  |  | 11(15.7) | 19(27.1) |  |  |
| Tumor differentiation |  |  |  |  |  |  |  |  |
| Well | 17(6.5) | 7(8.2) | 0.799 | 0.671 | 7(10.0) | 6(8.6) | 0.599 | 0.741 |
| Moderate | 164(62.8) | 49(57.6) |  |  | 46(65.7) | 43(61.4) |  |  |
| Poor | 80(30.7) | 29(34.1) |  |  | 17(24.3) | 21(30.0) |  |  |
| **Tumor location** |  |  |  |  |  |  |  |  |
| Left | 128(49.0) | 56(65.9) | 7.544 | 0.023 | 42(60.0) | 47(67.1) | 1.920 | 0.383 |
| Right | 99(37.9) | 23(27.1) |  |  | 25(35.7) | 18(25.7) |  |  |
| Left and right | 34(13.0) | 6(7.1) |  |  | 3(4.3) | 5(7.1) |  |  |
| **Morphologic grape** |  |  |  |  |  |  |  |  |
| Mass-forming | 221(84.7) | 51(60.0) | 24.215 | <0.001 | 54(77.1) | 50(71.4) | 1.411 | 0.494 |
| Periductal infiltrating | 24(9.2) | 17(20.0) |  |  | 8(11.4) | 13(18.6) |  |  |
| Intraductal growth | 16(6.1) | 17(20.0) |  |  | 8(11.4) | 7(10.0) |  |  |
| Tumor size (cm) |  |  |  |  |  |  |  |  |
| ≤5.0 | 135(51.7) | 50(58.8) | 1.299 | 0.254 | 39(55.7) | 41(58.6) | 0.117 | 0.733 |
| >5.0 | 126(48.3) | 35(41.2) |  |  | 31(44.3) | 29(41.4) |  |  |
| Major vascular invasion |  |  |  |  |  |  |  |  |
| No | 217(83.1) | 69(81.2) | 0.173 | 0.678 | 58(82.9) | 56(80.0) | 0.189 | 0.664 |
| Yes | 44(16.9) | 16(18.8) |  |  | 12(17.1) | 14(20.0) |  |  |
| Microvascular invasion |  |  |  |  |  |  |  |  |
| No | 235(90.0) | 71(83.5) | 2.657 | 0.103 | 64(91.4) | 59(84.3) | 1.674 | 0.196 |
| Yes | 26(10.0) | 14(16.5) |  |  | 6(8.6) | 11(15.7) |  |  |
| Perineural invasion |  |  |  |  |  |  |  |  |
| No | 223(85.4) | 66(77.6) | 2.830 | 0.093 | 56(80.0) | 57(81.4) | 0.046 | 0.830 |
| Yes | 38(14.6) | 19(22.4) |  |  | 14(20.0) | 13(18.6) |  |  |
| **Liver capsule involvement** |  |  |  |  |  |  |  |  |
| No | 180(69.0) | 69(81.2) | 4.739 | 0.029 | 51(72.9) | 56(80.0) | 0.991 | 0.319 |
| Yes | 81(31.0) | 16(18.8) |  |  | 19(27.1) | 14(20.0) |  |  |
| AJCC 8th edition T stage |  |  |  |  |  |  |  |  |
| T_1a_/T_1b_ | 63(24.1) | 26(30.6) | 2.168 | 0.338 | 17(24.3) | 21(30.0) | 5.130 | 0.077 |
| T_2_ | 132(50.6) | 43(50.6) |  |  | 46(65.7) | 34(48.6) |  |  |
| T_3_/T_4_ | 66(25.3) | 16(18.8) |  |  | 7(10.0) | 15(21.4) |  |  |
| AJCC 8th edition N stage |  |  |  |  |  |  |  |  |
| N0 | 193(73.9) | 61(71.8) | 0.156 | 0.693 | 50(71.4) | 50(71.4) | 0.000 | 1.000 |
| N1 | 68(26.1) | 24(28.2) |  |  | 20(28.6) | 20(28.6) |  |  |
| AJCC 8th edition TNM stage |  |  |  |  |  |  |  |  |
| IA/IB | 119(45.6) | 45(52.9) | 1.534 | 0.464 | 36(51.4) | 36(51.4) | 1.133 | 0.567 |
| II | 40(15.3) | 10(11.8) |  |  | 12(17.1) | 8(11.4) |  |  |
| IIIA/IIIB/IV | 102(39.1) | 30(35.3) |  |  | 22(31.4) | 26(37.1) |  |  |

Supplemental Table 3. Demographics and clinical characteristics of patients with HBV-ICC and Stone-ICC before and after PSM

|  | Before PSM | | χ^2^ | *P* | After PSM | | χ^2^ | *P* |
| --- | --- | --- | --- | --- | --- | --- | --- | --- |
|  | HBV -ICC (%) | Stone-ICC (%) |  |  | HBV -ICC (%) | Stone-ICC (%) |  |  |
| **Sex** |  |  |  |  |  |  |  |  |
| Male | 63(61.8) | 33(38.8) | 9.767 | 0.002 | 16(43.2) | 15(40.5) | 0.056 | 0.814 |
| Female | 39(38.2) | 52(61.2) |  |  | 21(56.8) | 22(59.5) |  |  |
| **Age (year)** |  |  |  |  |  |  |  |  |
| ≤55 | 51(50.0) | 26(30.6) | 7.213 | 0.007 | 14(37.8) | 15(40.5) | 0.057 | 0.812 |
| >55 | 51(50.0) | 59(69.4) |  |  | 23(62.2) | 22(59.5) |  |  |
| **Obstructive jaundice** |  |  |  |  |  |  |  |  |
| No | 100(98.0) | 69(81.2) | 15.155 | <0.001 | 36(97.3) | 35(94.6) | 0.347 | 0.556 |
| Yes | 2(2.0) | 16(18.8) |  |  | 1(2.7) | 2(5.4) |  |  |
| AFP (ng/ml) |  |  |  |  |  |  |  |  |
| ≤7.0 | 77(75.5) | 63(74.1) | 0.046 | 0.829 | 29(78.4) | 28(75.7) | 0.076 | 0.782 |
| >7.0 | 25(24.5) | 22(25.9) |  |  | 8(21.6) | 9(24.3) |  |  |
| CEA (ng/ml) |  |  |  |  |  |  |  |  |
| ≤5.0 | 77(75.5) | 60(70.6) | 0.569 | 0.451 | 25(67.6) | 26(70.3) | 0.063 | 0.802 |
| >5.0 | 25(24.5) | 25(29.4) |  |  | 12(32.4) | 11(29.7) |  |  |
| **CA19-9(U/ml)** |  |  |  |  |  |  |  |  |
| ≤39.0 | 48(47.1) | 25(29.4) | 6.067 | 0.014 | 11(29.7) | 10(27.0) | 0.066 | 0.797 |
| >39.0 | 54(52.9) | 60(70.6) |  |  | 26(70.3) | 27(73.0) |  |  |
| CA125(U/ml) |  |  |  |  |  |  |  |  |
| ≤35.0 | 59(57.8) | 51(60.0) | 0.089 | 0.765 | 16(43.2) | 23(62.2) | 2.656 | 0.103 |
| >35.0 | 43(42.2) | 34(40.0) |  |  | 21(56.8) | 14(37.8) |  |  |
| **Child-Pugh Grade** |  |  |  |  |  |  |  |  |
| Grade A | 99(97.1) | 74(87.1) | 6.964 | 0.010 | 35(94.6) | 34(91.9) | 0.214 | 0.643 |
| Grade B | 3(2.9) | 11(12.9) |  |  | 2(5.4) | 3(8.1) |  |  |
| **Type of resection** |  |  |  |  |  |  |  |  |
| Wedge resection | 49(48.0) | 28(32.9) | 8.778 | 0.012 | 8(21.6) | 11(29.7) | 0.940 | 0.625 |
| Minor hepatectomy | 43(42.2) | 36(42.4) |  |  | 22(59.5) | 18(48.6) |  |  |
| Major hepatectomy | 10(9.8) | 21(24.7) |  |  | 7(18.9) | 8(21.6) |  |  |
| Tumor differentiation |  |  |  |  |  |  |  |  |
| Well | 4(3.9) | 7(8.2) | 1.683 | 0.431 | 3(8.1) | 2(5.4) | 2.690 | 0.261 |
| Moderate | 59(57.8) | 49(57.6) |  |  | 28(75.7) | 23(62.2) |  |  |
| Poor | 39(38.2) | 29(34.1) |  |  | 6(16.2) | 12(32.4) |  |  |
| **Tumor location** |  |  |  |  |  |  |  |  |
| Left | 36(35.3) | 56(65.9) | 17.367 | <0.001 | 23(62.2) | 23(62.2) | 0.000 | 1.000 |
| Right | 53(52.0) | 23(27.1) |  |  | 10(27.0) | 10(27.0) |  |  |
| Left and right | 13(12.7) | 6(7.1) |  |  | 4(10.8) | 4(10.8) |  |  |
| **Morphologic grape** |  |  |  |  |  |  |  |  |
| Mass-forming | 88(86.3) | 51(60.0) | 16.944 | <0.001 | 31(83.8) | 31(83.8) | 3.086 | 0.214 |
| Periductal infiltrating | 8(7.8) | 17(20.0) |  |  | 2(5.4) | 5(13.5) |  |  |
| Intraductal growth | 6(5.9) | 17(20.0) |  |  | 4(10.8) | 1(2.7) |  |  |
| Tumor size (cm) |  |  |  |  |  |  |  |  |
| ≤5.0 | 52(51.0) | 50(58.8) | 1.150 | 0.283 | 18(48.6) | 20(54.1) | 0.216 | 0.642 |
| >5.0 | 50(49.0) | 35(41.2) |  |  | 19(51.4) | 17(45.9) |  |  |
| Major vascular invasion |  |  |  |  |  |  |  |  |
| No | 91(89.2) | 69(81.2) | 2.426 | 0.119 | 31(83.8) | 29(78.4) | 0.352 | 0.553 |
| Yes | 11(10.8) | 16(18.8) |  |  | 6(16.2) | 8(21.6) |  |  |
| Microvascular invasion |  |  |  |  |  |  |  |  |
| No | 87(85.3) | 71(83.5) | 0.110 | 0.740 | 33(89.2) | 31(83.8) | 0.463 | 0.496 |
| Yes | 15(14.7) | 14(16.5) |  |  | 4(10.8) | 6(16.2) |  |  |
| **Perineural invasion** |  |  |  |  |  |  |  |  |
| No | 96(94.1) | 66(77.6) | 10.860 | 0.001 | 33(89.2) | 34(91.9) | 0.158 | 0.691 |
| Yes | 6(5.9) | 19(22.4) |  |  | 4(10.8) | 3(8.1) |  |  |
| **Liver capsule involvement** |  |  |  |  |  |  |  |  |
| No | 64(62.7) | 69(81.2) | 7.669 | 0.006 | 24(64.9) | 27(73.0) | 0.568 | 0.451 |
| Yes | 38(37.3) | 16(18.8) |  |  | 13(35.1) | 10(27.0) |  |  |
| AJCC 8th edition T stage |  |  |  |  |  |  |  |  |
| T_1a_/T_1b_ | 27(26.5) | 26(30.6) | 2.803 | 0.246 | 11(29.7) | 10(27.0) | 1.194 | 0.551 |
| T_2_ | 45(44.1) | 43(50.6) |  |  | 19(51.4) | 16(43.2) |  |  |
| T_3_/T_4_ | 30(29.4) | 16(18.8) |  |  | 7(18.9) | 11(29.7) |  |  |
| AJCC 8th edition N stage |  |  |  |  |  |  |  |  |
| N0 | 82(80.4) | 61(71.8) | 1.918 | 0.166 | 28(75.7) | 26(70.3) | 0.274 | 0.601 |
| N1 | 20(19.6) | 24(28.2) |  |  | 9(24.3) | 11(29.7) |  |  |
| AJCC 8th edition TNM stage |  |  |  |  |  |  |  |  |
| IA/IB | 42(41.2) | 45(52.9) | 3.348 | 0.188 | 19(51.4) | 17(45.9) | 0.535 | 0.765 |
| II | 20(19.6) | 10(11.8) |  |  | 6(16.2) | 5(13.5) |  |  |
| IIIA/IIIB/IV | 40(39.2) | 30(35.3) |  |  | 12(32.4) | 15(40.5) |  |  |
