# Supplementary figures and images for: Recurrence and prognosis in intrahepatic cholangiocarcinoma patients with different etiology after radical resection: a multi-institutional study
Source: BMC Cancer. 2022 Mar 26;22:329. doi: 10.1186/s12885-022-09448-w (PMC8962079; doi:10.1186/s12885-022-09448-w)

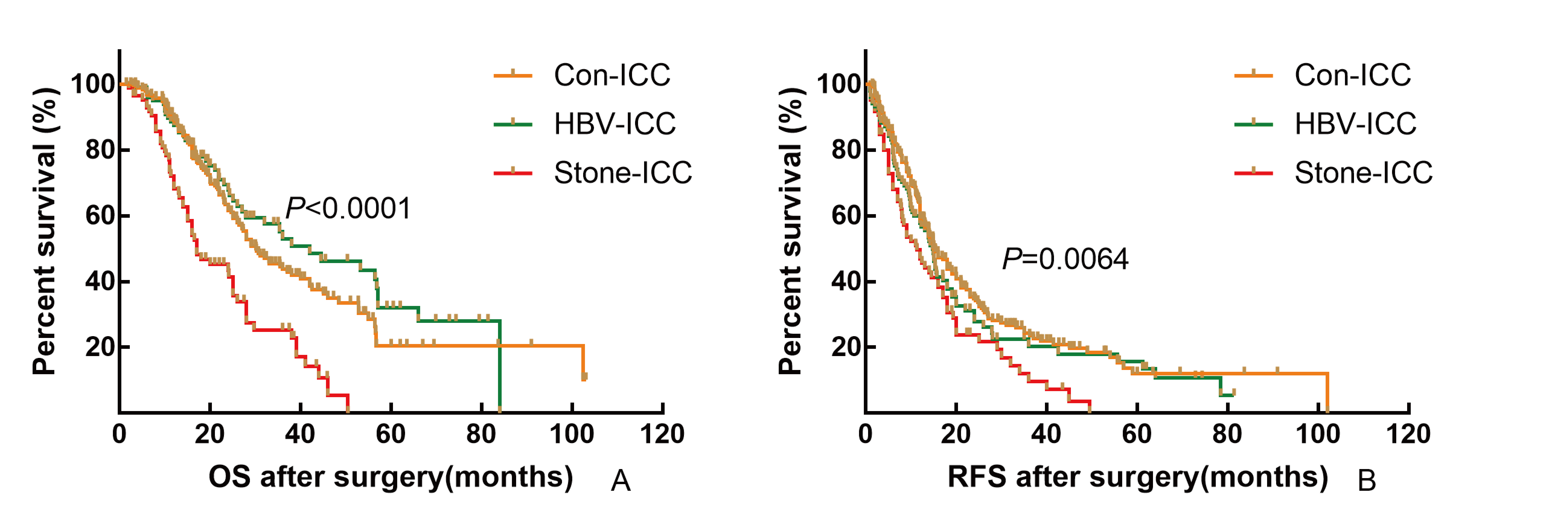

Supplement: Supplementary file 2 — Additional file 2: Supplement Fig 1. Comparison of overall survival and relapse-free survival after radical resection for conventional ICC, HBV-ICC and Stone-ICC (A and B). [file 12885_2022_9448_MOESM2_ESM.tif]
